# Elevating Interest and Expertise: Integrating Co-Design and Co-Production Into Foundation Year Development Initiatives During Psychiatry Rotations

**DOI:** 10.1192/bjo.2024.291

**Published:** 2024-08-01

**Authors:** Hassan Aquil Mallick, Mana Doyama, Itunuayo Ayeni, Isaac Obeng

**Affiliations:** CNWL, London, United Kingdom

## Abstract

**Aims:**

To provide a bespoke development program for foundation year (FY) trainees on rotation at CNWL NHS trust.

To increase interest in foundation trainees in choosing Psychiatry as a career.

Specific: 100% self-reported satisfaction with the FY development program by April 2024.

50% increase from baseline of self-reported interest in choosing psychiatry as a specialty by April 2024.

**Methods:**

We designed an online, 12 session teaching program for each 4 month cohort of foundation year doctors on rotation at St Charles Hospital, CNWL. We collect data at baseline, after each teaching session and at exit via online questionnaires. These are reviewed at PDSA meetings (including nominees from foundation cohort) by team quarterly. First cohort started in May 2023, we are currently in our 3rd cohort of this project. Each cohort has approximately 15 Foundation year Trainees. Our curricula integrates principles of Co-Design: 2 of the 12 teaching slot topics are voted by each cohort of foundation trainees. Co-Production: 2 of the 12 slots are always for a carer, service user or expert by experience. Bespoke: Each teacher is provided a written guide outlining the training grade of foundation trainees and highlights the needs for transferrable skills as FY trainees may not pursue psychiatry as a career. Quality Improvement: iterative learning from each cohort, with robust data collection methods (dedicated time set aside for feedback completion) and regular reviews by team. Teachers are canvassed via trust emails, trainee Whatsapp groups and patient liaison services at CNWL.

**Results:**

Cohort 1= no data collected. Data collection methods required improvement.

Cohort 2= data collection of 3 responses (23% completion rate). Data collected insufficient. Data collection method improved.

Cohort 3= data collection of 13 responses (87% completion rate).

Self-reported satisfaction with training program: 95.3% Interest in choosing psychiatry as a specialty: 30.4% at baseline to 76.2% at time of submission.

**Conclusion:**

This Foundation Year Development initiative provides a well-liked, bespoke and innovative approach to train foundation year doctors on placement at NHS trusts.

The majority of Foundation doctors (some surveys show 60%) are undecided on their specialty during foundation training and this is a unique opportunity to increase recruitment into psychiatry.

